# Gender differences in the associations between age trends of social media interaction and well-being among 10-15 year olds in the UK

**DOI:** 10.1186/s12889-018-5220-4

**Published:** 2018-03-20

**Authors:** Cara L. Booker, Yvonne J. Kelly, Amanda Sacker

**Affiliations:** 1 Institute for Social and Economic Research, University of Essex, Wivenhoe Park, Colchester, CO4 3SQ UK; 2 ESRC International Centre for Lifecourse Studies in Society and Health, Department of Epidemiology and Public Health, University College London, 1-19 Torrington Place, London, WC1E 6BT UK

**Keywords:** Adolescents, Gender, Growth curve modelling, Longitudinal studies, Social media interaction, Well-being

## Abstract

**Background:**

Adolescents are among the highest consumers of social media while research has shown that their well-being decreases with age. The temporal relationship between social media interaction and well-being is not well established. The aim of this study was to examine whether the changes in social media interaction and two well-being measures are related across ages using parallel growth models.

**Methods:**

Data come from five waves of the youth questionnaire, 10-15 years, of the Understanding Society, the UK Household Longitudinal Study (pooled *n* = 9859). Social media interaction was assessed through daily frequency of chatting on social websites. Well-being was measured by happiness with six domains of life and the Strengths and Difficulties Questionnaire.

**Results:**

Findings suggest gender differences in the relationship between interacting on social media and well-being. There were significant correlations between interacting on social media and well-being intercepts and between social media interaction and well-being slopes among females. Additionally higher social media interaction at age 10 was associated with declines in well-being thereafter for females, but not for males. Results were similar for both measures of well-being.

**Conclusions:**

High levels of social media interaction in early adolescence have implications for well-being in later adolescence, particularly for females. The lack of an association among males suggests other factors might be associated with their reduction in well-being with age. These findings contribute to the debate on causality and may inform future policy and interventions.

## Background

Rapid changes in technology have given rise to many important questions regarding their short- and long-term effects on overall health and well-being. Television viewing expanded people’s exposure to new and different cultures and ideas; however up until recently, it has not been an interactive medium. Thus it is especially important to explore, as this study does, whether there is a long-term relationship between interacting on social media and well-being among adolescents, as health-related behaviours and well-being levels track into adulthood [1–4]. The link between television viewing and health outcomes such as increased obesity, fasting insulin and other markers of metabolic risk has been well established leading many countries to establish guidelines for daily consumption [5]. More recently, technology has become more interactive, specifically with the advent of social media websites and smartphone apps. A recent report by the United Kingdom’s Office of Communications stated that adolescents aged 12-15 spend more time online than they do watching television [6]. Additionally, adolescents in the United Kingdom (UK) are ranked in the bottom third on overall well-being in a United Nations Children’s Fund report comparing several countries [7].

While social media allows for interaction between people, it is still a sedentary activity that can be done in a solitary environment. Conversely, social media are often used in group settings. Whether done in isolation or with friends, there may be risks to using social media, which could lead to poorer physical and mental health in adulthood [8, 9]. Risk factors such as social isolation [10], low self-esteem [11, 12], increased obesity [13] and decreased physical activity [14] may all contribute to later life health issues. While some studies have shown a negative relationship between interacting on social media and well-being, there are others which show positive associations. High quality interactions [15–17], reduced social isolation [16, 18] or information seeking [19] are all mechanisms through which well-being may be increased with social media use.

More recently, research has focused on the patterns of social media usage. There are different ways these patterns have been defined, Brandtzæg [16] identified five types, sporadics, lurkers, socializers, debaters and advanced. Others categorise users as active or passive [20–22]. As research into the effects of social media use and interaction has increased the theoretical framework underlying the relationship with well-being have continued to be developed. Verdyun et al., [22] suggest that the relationship operates differently for passive and active users. Active users may experiences an increase in social capital and connectedness resulting in an increase in well-being, however passive users may be more likely to experience upward social comparison leading to a reduction in well-being [22]. A review of current literature by Verduyn et al. [22] found mixed results for the passive mechanism while evidence for the active pathway was stronger.[22]While much of the early evidence linking social media interaction and well-being was based on cross-sectional data making causal inference impossible, evidence from longitudinal studies is increasing.

Recent longitudinal studies have reported longer term associations between social media interaction and well-being with mixed results [22–25]. In a study of Belgian adolescents, active private Facebook use, e.g. chatting or sending personal messages, was indirectly associated with lower depressed mood through increased perceived friend support and decreased avoidant coping [20]. Recent reviews of studies have analysed the associations between mental health and screen time or screen-based media [11, 22, 26]. One review included all forms of screen-based media and separated associations by type of mental health indicator [11]. They found support for a relationship of screen-based sedentary behaviours with increased depressive symptoms, increased inattention, hyperactivity problems, decreased self-esteem and decreased well-being and quality of life [11]. Evidence of a relationship with anxiety symptoms, internalising problems and eating disorder symptoms was inconclusive. [11] A meta-analysis examined evidence from cross-sectional and longitudinal studies separately with mixed findings. Among cross-sectional studies, the findings suggest a strong positive association between increased screen time and depression risk [26]. However among longitudinal studies’ findings suggest a negative, although non-significant association [26]. Further investigation of the longitudinal studies included identifying the quality of the studies, i.e. participant selection, measurement of constructs, methodology for addressing study design issues, control of confounding and appropriate statistical methodology. Therefore when lower quality studies were excluded increased screen time significantly predicted depression risk [26]. A limitation of these reviews is that there is a conflation, in some cases, of screen time with social media use or interaction on social media. Social media use is conducted using a screen, however there are features of social medial that cannot be found in traditional screen time such as television viewing [16].

A third recent review looked at two social media usage components, overall usage of social networking sites and types of social networking site use and their associations with subjective well-being [22]. They conclude that cross-sectional studies provide a mixed message on overall usage and subjective well-being, while longitudinal studies show more conclusively a decline in subjective well-being as a result of using social networking sites [22]. A limitation to this review is that the longitudinal studies sites used short follow-up times, one to two weeks, which may not translate into long-term effects. In their conclusions regarding types of social networking sites use and subjective well-being the authors suggest that passive use is associated with lower subjective well-being while most studies cited showed a positive association between active use and subjective well-being [22].

Prior research shows that screen-based media interaction increases whilst well-being levels decrease throughout adolescence and these changes differ by gender [6, 27, 28]. Many of the recent studies controlled for gender and age, where appropriate, but did not look at age or gender differences in screen-based media interaction or how associations with well-being might differ with age and gender. In the meta-analysis conducted by Liu et al., [26] gender and age moderation analyses were conducted which showed a significant positive association for males and adolescents under the age of 14; no significant associations were found for females or those over the age of 14. This suggests that there might be differences in the association between social media interaction and well-being by gender and across age groups.

The well-being measure used to examine the relationship between screen-based media and well-being might also be a factor which contributes to the diverse and sometimes conflicting results. Many studies have examined the associations between screen-based media and negative markers of well-being such as depression, socio-emotional difficulties and anxiety with mixed results [11, 20, 23, 29]. There have also been studies which have examined positive markers of well-being, such as happiness, self-esteem and quality of life, again with mixed results [11, 27]. Findings from a study of UK adolescents showed that interacting on social media for more than 4 h was associated with more socio-emotional difficulties, but not with lower levels of happiness suggesting that future research should investigate whether the relationship between social media interaction and positive and negative markers of well-being differs [27].

This study adds to the current literature by using longitudinal data from adolescents 10-15 years of age in the UK. The primary aim of this study is to examine changes in social media interaction and positive and negative markers of well-being with age and to determine whether any relationship exists between social media interaction and well-being trajectories. A secondary aim is to examine whether the social media interaction and well-being relationships and trajectories differ by gender. We also explore whether initial levels of well-being or social media interaction are predictive of rates of change in the other.

## Methods

### Participants

Respondents came from the youth panel of *Understanding Society: the UK Household Panel Study* (UKHLS). UKHLS is a nationally representative longitudinal study which interviews all household members annually (2009/10-2014/15). A stratified, clustered sampling scheme was used to identify primary sampling units. Additional information on the sampling scheme and data collection methods is available [30, 31]. All individuals 16 and older participated in the main survey while the youth questionnaire was given to adolescents aged 10-15. Youth panel members self-completed a paper-and-pencil survey. Verbal consent was required for participation for all respondents. Written consent is only required for requests to link administrative data to survey responses. Youth participation required the interviewer to ask the parent/guardian for their verbal consent, and receive an affirmative response, and then to ask the young person for their consent, at which point the young person was free to agree or refuse. Ethical approval was obtained from the University of Essex Ethics Committee and the Oxfordshire Research Ethics Committee (REC) A, REC reference OS/HO604/124.

In wave one, 4899 respondents participated in the youth panel, this represents 74% of the invited 6627 adolescents [32]. As children reach the age of 10 they are eligible to be included in the youth panel and at the age of 16 they are eligible to enter the adult interview. Over the first five waves of UKHLS, 9859 adolescents participated in the youth panel, participation in each wave ranged from a low of 3656 in wave 5 to a high of 5014 in wave 2. The number of adolescents who participated in just one wave was 3674; 2521 participated in two waves, 1874 in three, 1280 in four waves and only 510 have participated in all 5 waves. Males comprised 51% of the sample with 4990 individuals providing 11,073 person-age observations compared to 4869 females with 10,935 person-age observations.

### Measures

Social media interaction: Two questions were used to determine whether adolescents chatted via social media. The first question asked “Do you belong to a social web-site such as Bebo, Facebook or MySpace?” and the second question “How many hours do you spend chatting or interacting with friends through a social website like that on a normal school day?” Responses for the latter question were scored on a 5-point scale ranging from “none” to “7 or more hours.” Responses were then recoded so that those with no social networking profile were coded as “no profile” and other responses were recoded to “1 h or less”, “1-3 h” and “4 h or more” category.

Well-being: Happiness and socio-emotional difficulties reported by youth panel members were both used to examine whether social media interaction is differentially associated with positive and negative markers of well-being. Six questions covering different domains of life, i.e. friends, family, appearance, school, school work and life as a whole, were asked and scored on a 7-point Likert-type scale. Factor analysis confirmed that all questions loaded on to one factor, thus an overall happiness score was created with a range of 6-42 (Cronbach’s α = 0.77). Higher scores indicated higher levels of happiness [33].

Negative aspects of well-being were measured using the Strengths and Difficulties Questionnaire (SDQ). The SDQ is a validated instrument which screens for emotional and behavioural problems in children and adolescents aged 3-16 years [34]. The SDQ is comprised of 25 items; responses were ‘not true’, ‘somewhat true’ and ‘certainly true’. Twenty of these items covering hyperactivity/inattention, emotional symptoms, conduct problems and peer relationship problems are summed to create a total difficulties score which ranges from 0 to 40 (Cronbach’s α = 0.67). Higher scores on the total difficulties score indicate worse well-being. SDQ total difficulties scores of 20 or above indicate clinically relevant risk for mental problems [35]. This cut-off was chosen so roughly 90% of the sample would fall in the normal or borderline range and 10% would fall in the abnormal range [35]. The distribution of the SDQ total difficulties scores was slightly skewed, for both males (skewness = 0.56) and females (skewness = 0.53). Happiness questions are asked annually, however the SDQ is completed bi-annually.

Covariates: Control variables were chosen based on the literature and previous analysis, conducted on the same data, which showed independent associations between these variables and both screen-based media and well-being [6, 27, 28, 36]. Parent- and household-level covariates were included in this analysis. Marital status was included as the parent-level covariate while household-level covariates were highest educational attainment and household income. Covariates were also included in the models as time-varying or time-invariant, as appropriate. Ethnic group and mean household income were time-invariant while educational attainment and marital status were time-variant. The youth questionnaire only asked ethnic identity every other year, thus some adolescents may not answer these questions. Therefore we used the youth response to the ethnicity question where available, for the 19% (*n* = 1847) with no ethnicity parent’s reporting of their own ethnic group identity was used instead. Ethnic group was coded as White British, Black African/Caribbean, Asian, Other and Mixed. White British was the reference group.

At each wave, previous month net income is reported for the household. Household income was equivalised for household composition using the Organisation for Economic Co-operation and Development modified equivalence scale [37] and then log transformed to create a more normal distribution. Due to missingness and model convergence issues, income was averaged across all waves the young person participated in.

Each parent reported their highest education qualification at each wave. The highest reported qualification of either parent was used. Due to sample size in some of the categories, General Certificate of Secondary Education (GCSE) and other qualification were combined so that the categories were degree, other higher qualification, A-level, GCSE/Other qualification, no qualification; degree was the reference category. GCSE are exams taken at 16 years of age (school year 11) and A-levels are exam taken at 18 years of age (school year 13). Each parent also reported their marital or cohabitation status (referred to as partnership status) at each wave. Partnership status was dichotomised as partnered or not partnered with partnered as the reference category.

### Analysis

**Fig. 1:**
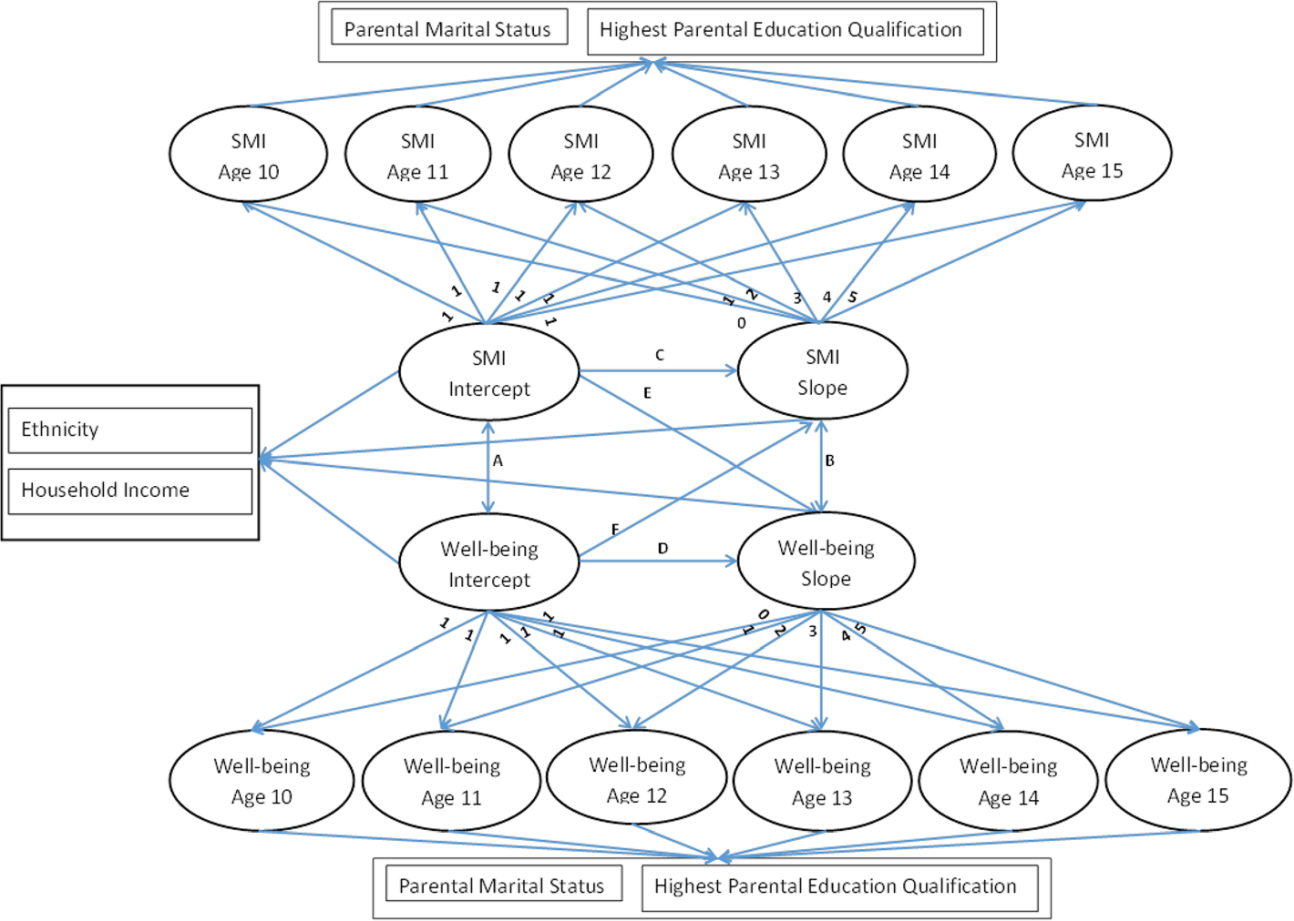
Conceptual parallel process growth model. Note SMI = Social Media Interaction; Double-headed arrows indicate correlations; Single-headed arrows indicate regression paths. Parameter A = correlation between social media interaction and well-being intercepts; Parameter B = correlation between social media interaction and well-being slopes; Parameter C = social media interaction slope regressed on social media interaction intercept; Parameter D = Well-being slope regressed on well-being intercept; Parameter E = well-being slope regressed on social media interaction intercept; Parameter F = social media interaction slope regressed on well-being intercept

We estimated parallel latent growth curve models using MPlus 7.3 [38] Well-being scores and social media interaction are repeated at each age and are modelled as distinct processes; the conceptual model is shown in Fig. 1. Rather than model over time, we modelled by age. Therefore these models do not measure change over time within individuals but rather change by age averaged across individuals [39]. We estimated four models two for happiness, one for females and one for males and two for SDQ total difficulties, one for females and one for males Linear growth parameters are estimated for each process giving an intercept and a slope. Factor loadings were fixed at zero at age 10, therefore the intercept is interpreted as either the well-being score or the amount of time spent using social media at age 10. Intercepts and slopes are allowed to covary across processes; additionally slopes of one process are regressed on the intercept of the other process to estimate the potential reciprocal influence of social media interaction and well-being as the panel members aged. All models controlled for the young person’s ethnic group, parental partnership status, highest educational attainment and mean household income. Highest educational attainment and marital status regression coefficients were set to be equal across age to estimate the average effects of each and to reduce any random fluctuations at each age. All adolescents aged 10-15 in a household were given the opportunity to complete a questionnaire; thus all models were adjusted for clustering within households.

## Results

The person-age distribution was similar and equal within each gender; each age group consisted of 16-17% of the overall sample (Table 1). A higher percentage of fathers than mothers reported being partnered rather than non-partnered, as resident fathers are more likely to participate than non-resident fathers. The majority of adolescents were White British (74%) with Asian as the second largest ethnic group (11% for males and 12% for females).

**Table 1:** Social Media Interaction, Well-being and Socio-Demographic Variable Descriptives for 10-15 Year Old UK Young People by Gender^a^

	Females							Males						
	Total	10 year olds	11 year olds	12 year olds	13 year olds	14 year olds	15 year olds	Total	10 year olds	11 year olds	12 year olds	13 year olds	14 year olds	15 year olds
	(*N* = 4869)	*n* = 1664	*n* = 1794	*n* = 1841	*n* = 1853	*n* = 1884	*n* = 1847	*N* = 4990	*n* = 1711	*n* = 1824	*n* = 1907	*n* = 1938	*n* = 1861	*n* = 1750
Social media interaction														
Do not have a profile/internet access	23	50	38	23	13	10	8	28	55	43	30	17	13	10
Less than 1 h per day	38	39	41	42	37	34	33	45	36	43	48	51	48	44
1-3 h per day	30	10	18	29	37	39	43	22	7	12	19	26	31	36
4 or more hours per day	10	1	3	7	13	17	16	5	2	2	2	6	8	10
Mother’s marital status (% partnered)	76	80	76	75	76	76	76	78	79	79	78	77	77	77
Father’s marital status (% partnered)	98	98	98	98	98	98	97	97	98	97	97	97	96	97
Highest parental qualification														
No qualification	13	11	12	13	14	14	15	13	13	12	12	13	13	14
Other qualification^b^	13	12	13	13	14	14	14	13	11	12	13	14	13	15
GCSE^c^	29	28	30	30	29	29	28	29	30	30	29	28	29	28
A-level^d^	17	19	18	17	17	16	16	18	17	19	19	17	18	17
Other higher qualificaiton^e^	11	12	10	11	11	11	11	11	12	11	11	11	11	11
Degree	16	18	17	17	16	16	15	16	16	16	16	16	16	16
Ethnicity														
White British	74							74						
Black African/Caribbean	6							5						
Asian	11							12						
Other	4							4						
Mixed	5							5						
Mean log household income	7.14 (0.43)							7.14 (0.43)						
Happiness scale	35.03 (5.14)	36.94 (4.32)	36.62 (4.42)	35.61 (4.88)	34.38 (5.17)	33.56 (5.47)	33.33 (5.24)	35.27 (4.83)	36.02 (4.63)	35.87 (4.71)	35.53 (4.73)	35.04 (4.85)	34.67 (5.04)	34.55 (4.84)
SDQ total difficulties	10.61 (5.60)	11.30 (5.74)	9.83 (5.42)	10.33 (5.61)	10.62 (5.59)	11.35 (5.83)	11.15 (5.28)	10.65 (5.69)	11.51 (5.89)	11.05 (6.03)	10.59 (5.66)	10.40 (5.63)	10.16 (5.37)	10.25 (5.43)

Abbreviation: GCSE, General Certificate of Secondary Education; SDQ, Strengths and Difficulties Questionnaire; UK, United Kingdom

^a^Social media Interaction, parental marital status, parental qualification and parental ethnicity are percentages; Mean log household income, happiness and SDQ total difficulties are means and standard deviations

^b^Other qualifications include CSE, skills certifications, apprenticeships, clerical qualifications, etc.;

^c^GCSE = exams taken at age 16 (year 11)

^d^A Level exam taken at age 18 (year 13)

^e^Examples of other higher qualifications are teaching, nursing or diploma certifications /qualifications

Table 1 shows that interacting on social media increased with age for both males and females. Females used social media more than males, a pattern that continued throughout adolescence. At age 13, half of females were chatting for more than 1 h per day, compared to one third of males. By the age of 15, 59% of females and 46% of males were chatting for 1 or more hours per day.

Well-being scores also differed by gender and age. Happiness scores decreased for females from a high of 36.94 (95% Confidence Interval [95% CI] = 36.73, 37.15) at age 10 to 33.33 (95% CI = 33.10, 33.57) at age 15. In this sample, young women with clinically relevant SDQ scores had a happiness level 6.95 (95% CI = 6.31, 7.58) points lower than young women who did not have clinically relevant SDQ total difficulties scores, 1.42 of a happiness standard deviation. The 3.44 (95% CI = 3.00, 3.89) point difference in happiness between female 10- and 15-year olds is 0.70 of the total female happiness standard deviation across all ages. With the exception of the difference between ages 10 and 11 and ages 14 and 15, all levels of happiness were significantly different from each other. Males showed a similar, albeit smaller, reduction in happiness levels going from 36.02 (95% CI = 35.80, 36.24) at age 10 to 34.55 (95% CI = 34.33, 34.78) at age 15. This is equivalent to 0.30 standard deviates on the happiness scale or one-quarter of the difference between young males with clinically and non-clinically relevant SDQ scores. Young males aged 13 and older were significantly less happy than both 10 and 11 year olds while 12 year olds were significantly happier than 14 and 15 year olds. SDQ scores decreased for males, but increased for females. At age 10 the average SDQ score was 10.30 (95% CI = 9.94-10.66) and rose to 11.15 (95% CI = 10.83-11.46) at age 15. Average female SDQ scores were significantly higher at ages 14 and 15 than the scores at age 10, 11 and 12. Conversely, males had an average SDQ score of 11.51 (95% CI = 11.15, 11.87) at age 10 which decreased to 10.25 (95% CI = 9.92, 10.59) at age 15. SDQ scores of males at ages 10 and 11 did not differ from each other but were significantly higher than the average scores of males aged 13, 14 and 15. While the average score at age 10 was higher than the age 12, there was no difference between the average age 11 and age 12 scores.

Significant differences between genders at specific ages were also observed. Ten and eleven year old females were significantly happier and had lower SDQ scores than males. These differences became non-significant at age 12 and at age 13 males reported higher levels of happiness while the SDQ scores were non-significantly different. Fourteen and fifteen year old males on average were significantly happier and had lower SDQ scores than females.

### Parallel growth model growth factor associations

The parameter estimates for the model intercepts, slopes and growth factor associations are given in Table 2. There were significant differences in the models between males and females. In both the happiness and SDQ models, there were significant correlations between the intercept of social media interaction and the intercept of each marker of well-being for females (Fig. 1, parameter A). These findings indicate that increased social media interaction was correlated with lower levels of happiness and higher levels of socio-emotional difficulties at age 10. While the happiness and social media interaction intercepts were uncorrelated in males, there was a significant correlation between the two intercepts in the SDQ model, Correlation parameter (r) =0.10 (95% CI = 0.01, 0.19). Parameter B, the correlation between the slopes of social media interaction and well-being was significant for females only. In both cases, an increase in social media interaction was correlated with a decline in happiness, *r* = − 0.23 (95% CI = − 0.36, − 0.09) and an increase in SDQ score, *r* = 0.26 (95% CI = 0.09, 0.43).

**Table 2:** Parameter estimates

	Happiness				SDQ total difficulties			
	Females (*n* = 4765)		Males (*n* = 4903)		Females (*n* = 4762)		Males (*n* = 4901)	
	PE	95% CI	PE	95% CI	PE	95% CI	PE	95% CI
Model Intercepts								
Well-being Intercept	36.55	(33.32, 39.78)	38.40	(35.20, 41.61)	13.57	(8.86, 18.27)	10.44	(5.54, 15.34)
Well-being Slope	1.65	(− 0.01, 3.31)	0.76	(−1.00, 2.52)	1.69	(0.44, 2.93)	0.78	(− 0.40, 1.95)
SMI intercept	0.00		0.00		0.00		0.00	
SMI slope	0.44	(− 0.32, 1.20)	0.47	(− 0.26, 1.20)	− 0.17	(− 0.71, 0.36)	0.11	(− 0.46, 0.68)
Growth factor associations								
Path A: Intercept SMI < −-> Intercept WB^a^	− 0.10	(− 0.19, − 0.01)	−0.02	(− 0.10, 0.07)	0.18	(0.08, 0.27)	0.10	(0.01, 0.19)
Path B: Slope SMI < −-> Slope WB^a^	− 0.23	(−0.36, − 0.09)	−0.02	(− 0.15, 0.12)	0.26	(0.09, 0.43)	0.17	(−0.03, 0.36)
Path C: Slope SMI < −- Intercept SMI	−0.08	(− 0.13, − 0.04)	−0.14	(− 0.17, − 0.11)	−0.08	(− 0.13, − 0.04)	−0.14	(− 0.17, − 0.11)
Path D: Slope WB < −- Intercept WB	−0.08	(− 0.12, − 0.04)	−0.07	(− 0.11, − 0.03)	−0.10	(− 0.15, − 0.05)	−0.08	(− 0.12, − 0.03)
Path E: Slope WB < −- Intercept SMI	−0.06	(− 0.13, 0.01)	−0.004	(− 0.05, 0.04)	0.10	(0.004, 0.19)	0.03	(−0.03, 0.10)
Path F: Slope SMI < −- Intercept WB	−0.01	(− 0.03, 0.001)	−0.01	(− 0.02, 0.002)	0.01	(− 0.01, 0.02)	− 0.001	(− 0.01, 0.01)
Model fit								
Loglikelihood	−43,001.75		−42,831.53		−31,170.95		−31,064.01	
AIC	86,111.50		85,771.06		62,449.90		62,236.02	
BIC	86,289.24		86,121.94		62,799.19		62,586.86	

*Abbreviations:* *AIC* Akaike Information Criterion, *BIC* Bayesian Information Criterion, 95% *CI* 95% Confidence Interval, *PE* Raw parameter estimate, *SMI* Social Media Interaction, *WB* Well-being; <−- regression; <−-> correlation

^a^Coefficients are correlations

For both males and females, the intercept of social media interaction was associated with the social media slope (Parameter C) and the well-being intercept was associated with the well-being slope (Parameter D). The associations were negative for both happiness and SDQ total difficulties. These findings indicate that adolescents with high levels of social media interaction at age 10 have less steep trajectories (slower rate of change) with age than those who interacted less social media at age 10. The happiness model correlation estimate for males is *r* = − 0.14 (95% CI = − 0.17, − 0.11) and females is *r* = − 0.08 (95% CI = − 0.13, − 0.04). Parameter estimates for the SDQ model were similar (Table 2). Similarly, high levels of happiness or a low level of socio-emotional difficulties at age 10 were associated with smaller changes in the respective marker of well-being with age (Parameter D).

Finally, there was only one significant association for Parameter E, the association between the social media interaction intercept and the SDQ slope. For females, increased interaction on social media at age 10 was associated with greater increases in SDQ with age, path coefficient = 0.10 (95% CI = 0.004, 0.19). The association approached significance (*p*-value = 0.07) in the happiness model for females, coefficient = − 0.06 (95% CI = − 0.13, 0.01). There were no significant associations for Parameter F, the slope of social media interaction regressed on the well-being intercept, however in the happiness models the female (*p*-value = 0.07) association approached significance.

### Parallel growth model covariate parameter estimates

Table 3 provides the associations of the covariates with the well-being and social media variables. There was no association between parental education and happiness for females. However lower levels of parental education were associated with lower levels of happiness for males. In the SDQ models, there was a dose-response relationship between parental education and their child’s SDQ. In both the happiness and SDQ models, all levels of parental educational attainment were associated with increased social media interaction for both males and females compared to adolescents whose highest parental achievement was at degree level. Having an unpartnered mother was associated with lower well-being for both males and females. Compared to adolescents who lived with a partnered mother, those living with an unpartnered mother interacted on social media more; the effect size was the same for both males and females in the happiness and the SDQ models. Living with an unpartnered father was associated with worse well-being for females only; there were no significant associations for males.

**Table 3:** Covariate parameter estimates^a,b^

	Happiness		SDQ total difficulties	
	Females (*n* = 4765)	Males (*n* = 4903)	Females (*n* = 4762)	Males (*n* = 4901)
Well-being^c^				
No qualification	0.24 (− 0.22, 0.69)	− 0.47 (− 0.90, − 0.03)	0.71 (0.10, 1.30)	1.50 (0.91, 2.10)
GCSE/Other qualification	0.11 (− 0.24, 0.46)	− 0.40 (− 0.73, − 0.06)	0.60 (0.15, 1.05)	0.90 (0.44, 1.36)
A-level	− 0.21 (− 0.66, 0.20)	− 0.29 (− 0.67, 0.08)	0.43 (− 0.09, 0.95)	0.80 (0.27, 1.32)
Other higher Qualification	0.02 (− 0.43, 0.46)	− 0.52 (− 0.97, − 0.08)	0.21 (− 0.38, 0.80)	0.45 (− 0.15, 1.04)
Degree (Ref)				
Unpartnered Mother	− 0.81 (− 1.12, − 0.51)	− 0.69 (− 0.98, − 0.39)	0.51 (0.12, 0.91)	0.88 (0.47, 1.29)
Partnered Mother (Ref)				
Unpartnered Father	−1.52 (−2.52, − 0.52)	− 0.60 (− 1.35, 0.15)	1.37 (0.23, 2.49)	0.77 (− 0.28, 1.81)
Partnered Father (Ref)				
Social Media Interaction^c^				
No qualification	0.85 (0.60, 1.10)	0.87 (0.62, 1.12)	0.85 (0.59, 1.11)	0.88 (0.61, 1.14)
GCSE/Other qualification	0.84 (0.65, 1.03)	0.62 (0.43, 0.80)	0.85 (0.66, 1.04)	0.62 (0.42, 0.81)
A-level	0.56 (0.33, 0.78)	0.59 (0.38, 0.81)	0.57 (0.35, 0.79)	0.60 (0.38, 0.82)
Other higher Qualification	0.60 (0.36, 0.84)	0.45 (0.22, 0.68)	0.61 (0.37, 0.85)	0.45 (0.22, 0.69)
Degree (Ref)				
Unpartnered Mother	0.41 (0.26, 0.56)	0.32 (0.17, 0.48)	0.41 (0.26, 0.56)	0.32 (0.16, 0.48)
Partnered Mother (Ref)				
Unpartnered Father	0.12 (− 0.37, 0.61)	0.37 (− 0.06, 0.80)	0.15 (− 0.34, 0.63)	0.37 (− 0.07, 0.82)
Partnered Father (Ref)				
Well-being Intercept				
Black African/Caribbean	1.06 (0.38, 1.74)	1.14 (0.25, 2.04)	−1.72 (−2.65, − 0.79)	−1.38 (−2.66, − 0.11)
Asian	0.46 (− 0.16, 1.07)	0.80 (0.25, 1.35)	− 0.91 (− 1.75, − 0.08)	−1.56 (− 2.39, − 0.72)
Other Ethnicity	0.84 (0.003, 1.68)	− 0.15 (− 1.08, 0.79)	− 0.37 (− 1.62, 0.88)	− 0.37 (− 1.62, 0.89)
Mixed Ethnicity	− 0.67 (− 1.55, 0.20)	0.42 (− 0.34, 1.17)	−0.10 (− 1.16, 0.96)	−0.83 (− 1.80, 0.14)
White (Ref)				
Mean Log Household Income	0.11 (−0.33, 0.54)	−0.28 (− 0.71, 0.16)	−0.57 (− 1.20, 0.07)	0.04 (− 0.63, 0.70)
Well-being Slope				
Black African/Caribbean	−0.05 (− 0.27, 0.17)	0.004 (− 0.25, 0.26)	−0.02 (− 0.26, 0.21)	−0.20 (− 0.51, 0.11)
Asian	0.18 (0.01, 0.36)	0.31 (0.15, 0.47)	−0.16 (− 0.37, 0.05)	−0.12 (− 0.34, 0.09)
Other Ethnicity	− 0.14 (− 0.41, 0.13)	0.14 (− 0.09, 0.36)	−0.28 (− 0.56, − 0.002)	−0.02 (− 0.31, 0.26)
Mixed Ethnicity	0.04 (− 0.24, 0.32)	−0.15 (− 0.37, 0.08)	0.08 (− 0.15, 0.32)	0.11 (− 0.13, 0.34)
White (Ref)				
Mean Log Household Income	0.06 (−0.06, 0.17)	0.20 (0.10, 0.31)	−0.03 (− 0.17, 0.12)	−0.03 (− 0.18, 0.11)
Social Media Interaction Intercept				
Black African/Caribbean	−0.04 (− 0.44, 0.36)	0.07 (− 0.53, 0.68)	−0.04 (− 0.44, 0.36)	0.06 (− 0.54, 0.67)
Asian	−0.81 (−1.14, − 0.48)	−0.47 (− 0.80, − 0.13)	−0.80 (− 1.13, − 0.47)	−0.48 (− 0.81, − 0.14)
Other Ethnicity	−0.04 (− 0.50, 0.42)	0.22 (− 0.27, 0.71)	−0.01 (− 0.47, 0.45)	0.21 (− 0.28, 0.70)
Mixed Ethnicity	−0.53 (− 1.00, − 0.06)	−0.08 (− 0.57, 0.41)	−0.53 (− 1.00, − 0.07)	−0.09 (− 0.57, 0.40)
White (Ref)				
Mean Log Household Income	−0.34 (− 0.58, − 0.10)	−0.16 (− 0.41, 0.09)	−0.36 (− 0.60, − 0.12)	−0.16 (− 0.41, 0.09)
Social Media Interaction Slope				
Black African/Caribbean	−0.05 (− 0.17, 0.06)	−0.02 (− 0.14, 0.10)	−0.06 (− 0.17, 0.06)	−0.03 (− 0.15, 0.09)
Asian	− 0.27 (− 0.37, − 0.17)	−0.10 (− 0.18, − 0.03)	−0.27 (− 0.37, − 0.17)	−0.11 (− 0.19, − 0.04)
Other ethnicity	−0.12 (− 0.26, 0.01)	−0.10 (− 0.20, 0.004)	−0.17 (− 0.27, − 0.03)	−0.09 (− 0.19, 0.01)
Mixed ethnicity	0.13 (0.01, 0.26)	0.01 (−0.09, 0.11)	0.14 (0.02, 0.27)	0.01 (−0.09, 0.10)
White (Ref)				
Mean log household income	0.08 (0.02, 0.14)	0.05 (−0.004, 0.11)	0.09 (0.03, 0.15)	0.06 (−0.002, 0.11)

*Abbreviation:* *GCSE* General Certificate of Secondary Education, *SDQ* Strengths and Difficulties Questionnaire

^a^Other ethnicity = Gypsy or Irish Traveller, Other White, Any other Asian background, Any other Black background, Arab, Any other ethnic group

^b^GCSE exams taken at age 16 (year 11); A Level exam taken at age 18 (year 13); Examples of other higher qualifications are teaching, nursing or diploma certifications /qualifications; Other qualifications include CSE, skill s certifications, apprenticeships, clerical qualifications, etc.

^c^Average effects across age - covariate regressions were held to be equal across ages

Time-invariant associations were mixed and should be interpreted with caution due to the aggregation of ethnic groups. Black African/Caribbean adolescents had better well-being at age 10 compared to White British adolescents. Asian (Indian, Bangladeshi or Pakistani) males had higher levels of happiness at age 10 and both Asian males and females showed a greater increase in happiness with age when compared to their White British counterparts. Asian adolescents also had lower levels of socio-emotional difficulties at age 10 compared to White British adolescents. Asian adolescents used social media less at age 10 and their increase in use with age was slower than for White British adolescents. In the SDQ model, males from higher income households had greater increases of happiness with age compared to those from lower income households. In both well-being models, females in higher income households interacted on social media less at age 10; however their interaction increased more from 10 to 15 years more than adolescents in lower income households.

## Discussion

The results from this study showed that social media interaction increases with age and happiness decreases with age for both males and females. While socio-emotional difficulties decreased with age for males, they increased for females. The parallel growth models showed stark differences by gender, although the patterns were similar between the two measures of well-being. Worse well-being was associated with greater social media interaction at age 10 and the changes over time were also associated for females. Of most importance, greater interaction on social media at age 10 was associated with worsening socio-emotional difficulties with age among females. The findings for males showed that social media interaction and levels of well-being at age 10 were associated with their changes with age; however there were no cross-associations. Meaning that initial levels of well-being or social media interaction was not associated changes in interacting on social media or well-being levels, respectively. Only, social media interaction and SDQ scores were associated at age 10 in the SDQ model.

The findings indicate that well-being at older ages among females is associated with how much they interacted on social media at age 10; this was not the case for males. This is one of the first studies to show such stark differences between social media interaction and well-being between males and females. Many studies control for gender and do observe a significant gender main effect; however they do not test for gender interactions or stratify by gender [25, 40, 41]. In a cross-sectional analysis of UK adolescents, Brodersen et al., [42] found that the emotional symptoms subscale of the SDQ was associated with sedentary behaviour for females but not for males. Verduyn et al. [22] have offered potential pathways through which active and passive social media interaction may impact well-being, social capital and upward comparison. It is possible that as adolescent females age there is an increase in upward social comparison leading to decreases in well-being. While Verduyn et al. [22] do not theorise on the effects of active use on upward social comparison, it is possible active use is also associated with upward social comparison. Thus there may be a mediating role of upward social comparison on the relationship between social media interaction and well-being among females as they age. It is possible that by only controlling for gender and looking across age, these studies are masking the true relationships between social media interaction and well-being as they might differ by gender.

The male models did show that both happiness and socio-emotional difficulties decreased with age, however if these reductions are not associated with social media interaction what other factors could be responsible? Many studies have shown that social media interaction is higher among females than males while males are more likely to participate in gaming, either via computer or console [25, 27, 41, 42]. As gaming has become as interactive as social media, it is possible that greater associations between gaming and well-being might be found for males than females. Preliminary analysis on this sample suggests this, data not shown.

The personal and household characteristics of the adolescents produced interesting findings. Levels of well-being were better among Black African/Caribbean and Asian adolescents and changes in happiness were greater in Asians. This finding of better well-being of ethnic minority adolescents in the UK has been found elsewhere [43, 44]. A new finding of this study is that Asian adolescents chatted on social media less and their increase with age was lower than White British adolescents. Finally there was an association between social position and social media interaction in that adolescents from households with lower education or income had higher levels of interacting on social media and among females lower income was associated with more social media interaction at age 10, which has been replicated with US adolescents [45] but not in the UK [46].

There are several strengths of this study. It uses longitudinal data from a nationally representative sample. We were able to estimate models separately by gender showing significant differences in growth factor associations. This study controlled for several time-invariant and time-varying covariates. The associations between those covariates and the intercept and slopes of social media interaction and well-being intercepts and slopes differed. Associations also varied by age. Finally, the questions included in this study only assess one form of active social media interaction, i.e. chatting, and does not assess other forms of active interaction, nor passive interaction. So while we cannot examine differences between active and passive use, we are able to look at longitudinal effects of active use. Active interaction implies content contribution or creation while passive interaction includes reading but not commenting on posts. Thus active social media interaction may lead to increased feelings of connectedness and thus better well-being. The findings from this study contradict this hypothesis as well as previous findings [22]. There are limitations, however, the social media question asks specifically about interaction on a normal school day and not social media interaction during the weekend or when not in school, which might be higher. Thus the findings may be underestimated. Additionally, there are no questions on patterns or reasons that adolescents interact with social media. Recent studies have identified typologies of use and have examined how patterns of use are associated with well-being. [16, 20–22]. Future waves of UKHLS ask about weekend use and should be compared to weekday use. While UKHLS is longitudinal, it was not possible to use parallel latent growth curve models to examine within individual changes in the social media interaction and well-being relationship due to the replacement nature of the youth questionnaire and the long data collection period, 2 years, which did not allow for creation of cohorts. Use of a longitudinal study without these issues should enable further examination of changes over time within individuals.

## Conclusions

Advances in technology have resulted in increases in sedentary behaviour and, in the past, solitary activities. However with the creation of social media it is possible to interact with others while still being separate. Adolescents are increasingly engaged in social media and the long-term effects on well-being are not fully known. Some studies suggest that interacting on social media might reduce social isolation; however there are others which have come to opposite conclusions. The findings of this study show gender differences in that greater social media interaction at age 10 was associated with lower levels of well-being at later ages among females. The lack of significant associations among males, suggests that other factors are associated with the reduction of well-being during adolescence. Future studies should examine what these factors could be.

Social media interaction increases with age during adolescence and the current generation is not expected to reduce their use once they enter adulthood. It is therefore important to educate adolescents, specifically females, and their parents on the consequences of high levels of use at young ages on their future well-being, not just in later adolescence but in adulthood as well.

## Abbreviations

CI: Confidence Interval
GCSE: General Certificate of Secondary Education
SDQ: Strengths and Difficulties Questionnaire
UK: United Kingdom
UKHLS: UK Household Longitudinal Study
